# Use of a novel biliary stent to prevent distal stent migration in benign anastomotic stricture

**DOI:** 10.1055/a-2067-4442

**Published:** 2023-04-21

**Authors:** Hirokazu Saito, Yoshitaka Kadowaki, Atsushi Fujimoto, Kana Ohmoto, Shuji Tada

**Affiliations:** Department of Gastroenterology, Kumamoto City Hospital, Kumamoto, Japan

Endoscopic treatment using balloon enteroscopy-assisted endoscopic retrograde cholangiopancreatography (ERCP) is useful for hepaticojejunostomy anastomotic strictures. Although biliary stent placement is conducted to avoid stricture recurrence or realize stricture resolution after balloon dilation 1 2 , distal stent migration is a common problem 3 4 .

An 11-year-old-boy, who had undergone hepaticojejunostomy for pancreaticobiliary maljunction 1 year earlier, underwent single-balloon endoscope-assisted ERCP to resolve a benign anastomotic stricture ( Fig. 1 a ). After balloon dilation, a 7-Fr straight-type plastic stent was placed into the intrahepatic bile duct to prevent stricture recurrence ( Fig. 1 b ). However, distal stent migration occurred at 4 weeks after the procedure.

**Fig. 1:**
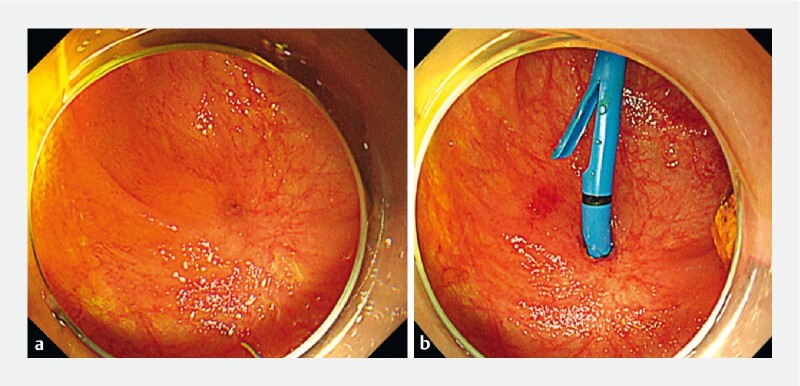
Endoscopic images. **a** After hepaticojejunostomy for pancreaticobiliary maljunction, a benign anastomotic stricture was noticed. **b** A 7-Fr straight-type plastic stent was placed into the intrahepatic bile duct after balloon dilation.

We used a novel 7-Fr Tanenbaum-type biliary stent integrated with a nasobiliary drainage catheter (UMIZAS NB STENT; Olympus Medical System, Tokyo, Japan) to prevent distal stent migration ( Fig. 2 a ). As the attached pusher catheter was too short to place the stent using a short-type enteroscope (SIF-H290S; Olympus Medical System), the outer sheath of a snare designed for the colon (Snare Master; Olympus Medical System) was used as the pusher catheter. First, we cut the outer sheath of the snare at the proximal side ( Fig. 2 b , Video 1 ) and covered the nasobiliary drainage catheter with the outer sheath of the snare after withdrawing the associated pusher catheter. The inner nasobiliary catheter protruded approximately 15 mm from the tip of the stent, and the outer sheath and nasobiliary catheter were fixed with tape. The nasobiliary catheter and outer sheath were removed after the 7-Fr biliary stent was placed into the intrahepatic duct ( Fig. 2 c ). After 3 months, there was no evidence of distal stent migration.

**Fig. 2:**
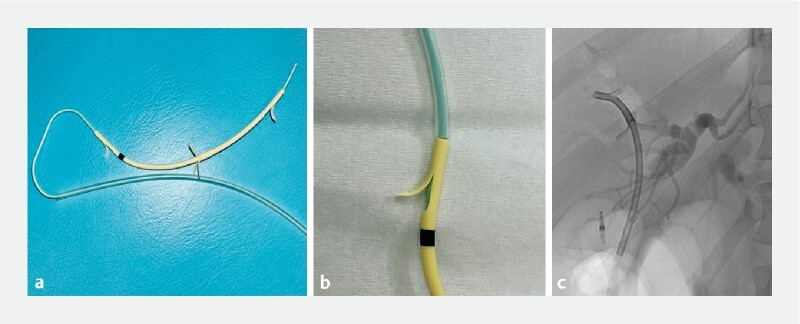
The novel biliary stent. **a** Novel 7-Fr Tanenbaum-type stent integrated with a nasobiliary drainage catheter. However, when used with a short-type enteroscope, which has a length of 167 cm between the forceps channel and the tip of the scope, the effective length of the attached pusher catheter was only 155 cm, making it impossible to place the stent. **b** The outer sheath of a snare designed for the colon, which has an effective length of 220 cm, was cut at the proximal side to use as the pusher catheter. This outer sheath was 7.3 Fr and just fitted over the 7-Fr biliary stent. **c** Using a short-type enteroscope, the 7-Fr biliary stent was inserted into the intrahepatic duct.

**Video 1** Use of a novel outside biliary stent with the outer sheath of a snare designed for the colon to avoid distal stent migration for a benign hepaticojejunostomy anastomotic stricture.


To reduce distal stent migration, a unique 7-Fr biliary stent integrated with a nasobiliary drainage catheter using the outer sheath of a snare designed for the colon may be helpful in surgically altered anatomy.

Endoscopy_UCTN_Code_TTT_1AR_2AZ

## Notes

Endoscopy E-Videos

https://eref.thieme.de/e-videos
*E-Videos* is an open access online section of the journal *Endoscopy* , reporting on interesting cases and new techniques in gastroenterological endoscopy. All papers include a high-quality video and are published with a Creative Commons CC-BY license. Endoscopy E-Videos qualify for HINARI discounts and waivers and eligibility is automatically checked during the submission process. We grant 100% waivers to articles whose corresponding authors are based in Group A countries and 50% waivers to those who are based in Group B countries as classified by Research4Life (see: https://www.research4life.org/access/eligibility/ ). This section has its own submission website at https://mc.manuscriptcentral.com/e-videos
